# The Role and Mechanisms of Antimicrobial Peptides in Overcoming Multidrug-Resistant Bacteria

**DOI:** 10.3390/molecules30010128

**Published:** 2024-12-31

**Authors:** Jinhui Yang, Junning Zhang, Zeyu Feng, Yunqi Ma

**Affiliations:** School of Pharmacy, Binzhou Medical University, Yantai 264003, China; 19712052937@163.com (J.Y.); 15339970946@163.com (J.Z.); fzy2596592390@163.com (Z.F.)

**Keywords:** AMPs, MDR bacteria, antibiotic resistance, peptide engineering, clinical trials, antimicrobial drugs

## Abstract

Multidrug-resistant (MDR) bacteria are becoming more and more common, which presents a serious threat to world health and could eventually render many of the antibiotics we currently use useless. The research and development of innovative antimicrobial tactics that can defeat these hardy infections are imperative in light of this predicament. Antimicrobial peptides (AMPs), which have attracted a lot of attention due to their distinct modes of action and capacity to elude conventional resistance mechanisms, are among the most promising of these tactics. As a promising substitute for conventional antibiotics, AMPs are a varied class of naturally occurring compounds that target bacteria membranes and disrupt cellular activities to demonstrate broad-spectrum antimicrobial activity. The objective of this study is to present a thorough summary of the current knowledge regarding AMP mechanisms against MDR bacteria, including immunological modulation, interactions with microbial membranes, and possible synergy with currently used antimicrobial drugs. In addition, we define the review’s scope to include the most recent developments in AMP research, emphasizing the innovations’ development, optimization, and therapeutic promise. We hope to emphasize the crucial role that AMPs will play in the future of antimicrobial therapy by bringing together recent research and highlighting current issues. We also hope to advocate for AMPs’ continued research and development as part of a comprehensive strategy to counteract the growing threat of antibiotic resistance.

## 1. Introduction

Pathogens known as multidrug-resistant bacteria (MDROs) are resistant to several widely used antibiotics, rendering conventional treatments ineffective and posing a major risk to the public’s health worldwide [1,2]. These bacteria’s resistance to drugs is mostly due to two processes: gene mutation and horizontal gene transfer.

Random genetic alterations in the bacterial genome are referred to as gene mutations, and they can impact how sensitive the bacteria are to antibiotics. Certain bacteria have the ability to mutate in order to develop enzymes that break down the structure of antibiotics or to acquire outer membrane proteins that limit the entry of medications into cells [3]. Furthermore, when exposed to antibiotic stress, bacteria may initiate the so-called SOS stress response, which raises the frequency of mutations and may result in drug resistance [4].

Horizontal gene transfer involves the movement of drug resistance genes between different bacterial individuals. These genes can be transferred from one bacterium to another through mobile genetic elements such as plasmids, transposons, bacteriophages, etc., resulting in the rapid spread of drug resistance. This mechanism makes it possible for even bacteria that are sensitive to certain antibiotics to quickly develop resistance to them and form so-called “superbugs” [5,6].

Methicillin-resistant Staphylococcus aureus (MRSA) is one of the best known multidrug-resistant bacteria that is resistant to a variety of β-lactam antibiotics, including methicillin [7]. Vancomycin-resistant enterococci (VRE) and multidrug-resistant tuberculosis (MDR-TB) are also serious public health problems with resistance to vancomycin, a last-line antibiotic, and multiple anti-tuberculosis drugs, respectively [8,9].

The global distribution and prevalence of these multidrug-resistant bacteria has attracted worldwide attention. It is estimated that by 2050, drug resistance could cause more than 10 million deaths per year, surpassing cancer deaths to become one of the leading causes of death worldwide [10]. In the United States, for example, nearly 23,000 people die each year from antibiotic resistance, according to the Disease Control and Prevention (CDC) [11]. These statistics and examples underscore the seriousness and urgency of solving the problem of multidrug-resistant bacteria. Therefore, understanding the production mechanism of multidrug-resistant bacteria and their spread and impact on a global scale is very important for formulating effective control strategies.

AMPs are a diverse and ancient class of molecules that play a crucial role in the innate immune system of various organisms, from microorganisms to multicellular plants and animals, including humans [12]. These peptides provide a potential solution in the ongoing fight against MDR bacteria, which pose a significant threat to global health.

AMPs are small peptides composed of 7 to 100 amino acids that are naturally produced and released by cells as part of their defense mechanisms [13]. They play a crucial role in the first line of defense against invading pathogens, providing an immediate and broad-spectrum antimicrobial response. The production of AMPs can be triggered by various stimuli, including the presence of foreign microbes, tissue damage, or inflammation.

AMPs have a broad-spectrum activity, exhibiting antimicrobial properties against a wide range of pathogens, including Gram-positive and Gram-negative bacteria, fungi, enveloped and non-enveloped viruses, and even some cancer cells [14,15]. In the context of MDR bacteria, AMPs can be crucial in targeting and eliminating pathogens that have developed resistance to conventional antibiotics [16].

The mechanisms of action of AMPs are diverse and can include direct interactions with microbial cell membranes, leading to permeabilization and disruption [17,18,19]. Additionally, they can inhibit essential cellular processes such as protein synthesis and DNA replication, and they can modulate the host immune response. Some AMPs also have the ability to form pores in the microbial membrane, leading to cell lysis and death.

**Figure 1:**
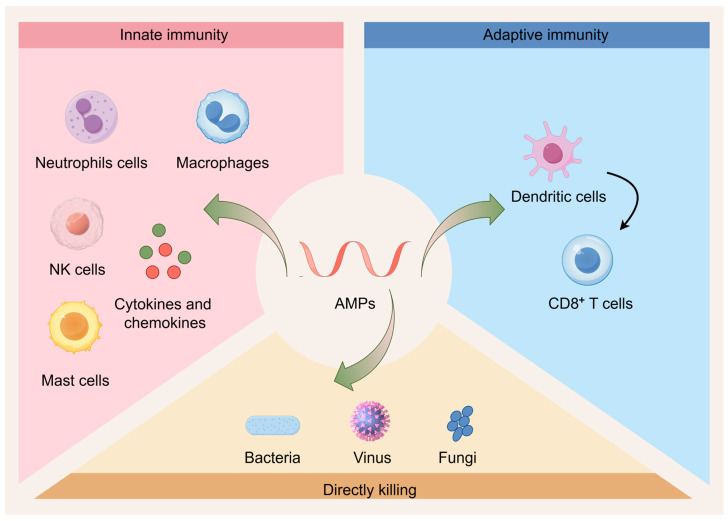
AMPs’ immune-regulating pathways. On the one hand, AMPs can stimulate immune cells in the natural immune system, including mast cells, NK cells, neutrophils, and macrophages; they can also cause the creation of cytokines (green balls) and chemokines (red balls), which in turn can engulf and destroy infections. However, by stimulating dendritic cells to deliver antigens to T cells and triggering the activation of cytotoxic T cells to eliminate infections, antimicrobial peptides can also trigger adaptive immune responses.

Understanding the mechanisms of action of AMPs is crucial for developing effective antimicrobial strategies against MDR pathogens. AMPs have a broad-spectrum activity against bacteria, fungi, and viruses, making them effective against MDR bacteria that have developed resistance to conventional antibiotics (Figure 1). The interaction between AMPs and bacterial membranes is unique and often differs from that of conventional antibiotics [20]. This provides a new approach to overcome existing resistance mechanisms.

Furthermore, current research is aimed at improving the therapeutic potential of AMPs. This involves enhancing their stability to prevent rapid degradation in biological environments, increasing their specificity to minimize off-target effects, and reducing toxicity to host cells [21,22,23].

The potential for AMPs to be used in combination therapies is an important area of research [24,25]. Research has shown that combining AMPs with conventional antibiotics can enhance the overall efficacy of treatment, as it may help to overcome resistance and increase the killing power against MDR bacteria [26,27]. This synergistic effect can lead to lower doses of antibiotics being required, which in turn reduces the risk of further resistance development.

The following sections of this review will examine the world of AMPs in more detail, including their various mechanisms, optimization strategies, and potential applications in combination therapies. This will highlight their crucial role in the ongoing fight against MDR pathogens [25]. They will fill the gap in the understanding of new antibacterial mechanisms and make up for the shortcomings of existing antibiotics in resisting multi-drug-resistant bacteria so as to provide more ideas for the development of antibacterial drugs and clinical treatment based on different principles.

## 2. Antimicrobial Peptides

**Figure 2:**
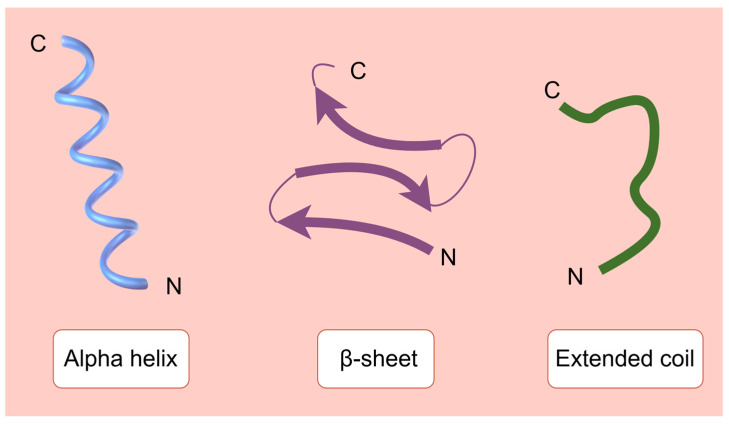
Simplified models of alpha helix, β-sheet, and extended coil structures.

In a broad range of organisms, AMPs are a varied family of naturally occurring chemicals that are essential to the innate immune system. AMPs can be roughly divided into four primary groups: α-helical, β-sheet, loop, and extended peptides, based on their structure and amino acid makeup (Figure 2).

Each family of AMP has different characteristics and mechanisms of action. Two noticeable families are defensins and cathelicidins.

Defensins are small, cysteine-rich peptides that are present in a variety of organisms and have been the subject of extensive research in humans. Defensins are divided into two main groups: α-defensins and β-defensins. They have the ability to disrupt microbial membranes, interfere with bacterial processes such as DNA replication and protein synthesis, and modulate the immune response [28,29,30]. One of the most efficient human AMPs is the human beta defensin-3 (hBD-3), which is produced by, e.g., keratinocytes and lung epithelial cells. The hBD-3 variant interacts with the outer membrane of Gram-negative bacteria through a mechanism similar to disordered circular pores, thereby killing bacteria [31].

Cathelicidins are larger peptides that contain a conserved cathelin pro-domain and a variable C-terminal antimicrobial domain [32]. The human cathelicidin (LL37) is known for its activity against a wide range of bacteria and its role in modulating immune responses [33].

Their evolutionary adaption to diverse environmental niches is reflected in this classification in addition to their structural diversity. A wide range of creatures are the source of amino acids macromolecules (AMPs), including humans (defensins and antibiotics), animals (magainin from frogs), plants (thionine), and even bacteria (bacteriocins) [12] (Table 1). The extensive range of life forms in which AMPs are found highlights their essential function in defense and survival, pointing to their potential as broad-spectrum antibiotics [34]. Researchers have discovered peptides with strong action against bacteria, fungi, viruses, and even cancer cells by examining the wide range of AMP sources [35]. These peptides offer a wealth of templates for creating new antimicrobial medicines.

The size, charge, and amphipathy of AMPs are among their general properties, and these factors play a major role in how they work [40,41]. Since most AMPs are small—typically containing 12–50 amino acids—they can interact with microbial membranes quickly. Fish antimicrobial peptides (AMPs) are small peptides, being considered critical as the first line of defense against a wide spectrum of pathogens. Beyond their antimicrobial function, fish AMPs possess several relevant but little-known characteristics and capabilities such as host-defense peptides, antioxidants, active compounds of immunogenic drugs and as adjuvants. Moreover, their antitumor properties make them potential drugs to be used in oncological treatments in human subjects [42].

All things considered, the special qualities and adaptable functions of AMPs highlight their importance as a key element of antimicrobial treatments of the future, justifying additional study and advancement to address existing obstacles and fulfill their therapeutic potential. It is evident that AMPs have attracted significant attention from the research community with considerable progress being made in the field. Furthermore, the advent of computer-assisted strategies has emerged as a potent and promising technological advancement for the precise prediction and design of novel AMPs. Table 2 shows the latest status of the design of new AMPs or peptide mimics.

## 3. Mechanisms of AMP Action Against Bacteria

### 3.1. Direct Mechanisms of Action

**Figure 3:**
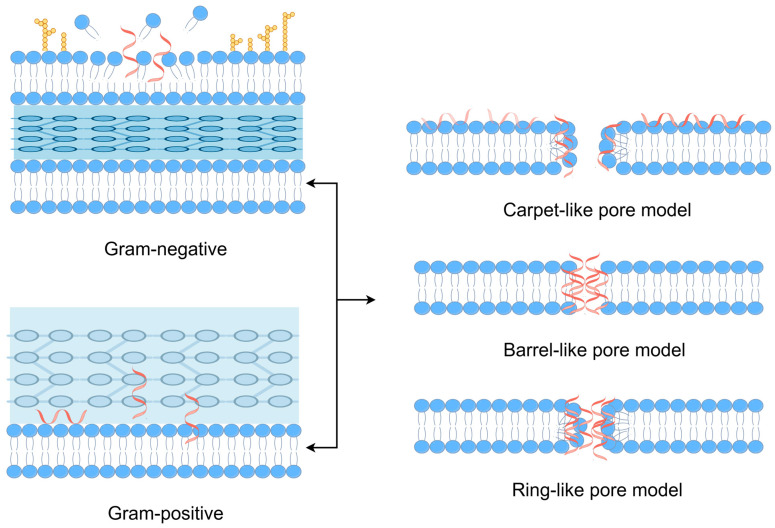
The destruction of bacterial membranes by AMPs. The role of AMPs is mainly based on their effect on the plasma membrane, that is, disturbance or destruction. However, in the presence of Gram-negative bacteria, AMPs first pass through the outer phospholipid membrane and then through the peptidoglycan layer to reach the intima. In Gram-positive bacteria, they cross the thick cell wall of peptidoglycan. They induce perturbations through pore formation in carpet-like, barrel-like or ring-like pore models (depending on peptide composition). Blue represents the bacterial membranes. Red represents antimicrobial peptides (AMPs) interacting with the membranes. Yellow represents specific molecular components unique to Gram-negative bacterial membranes.

The fight against bacteria that are resistant to drugs has led to a more thorough investigation of the mechanisms of action of AMPs, which has revealed a more comprehensive strategy for treating microbial infections. A key component of AMPs’ antibacterial action is their capacity to rupture and permeate bacterial membranes [20]. The primary reason for this behavior is that AMPs are amphipathic, which enables them to selectively interact with the lipid components of bacterial membranes [47,48]. When AMPs bind, they have the ability to enter into the membrane bilayer and generate pores that damage the membrane’s integrity (Figure 3). This can eventually cause cell lysis and the release of essential cellular contents.

Due to its ability to function independently of the metabolic pathways that are frequently the targets of conventional antibiotics and the hubs of resistance mechanisms, this membrane-targeted mechanism is very efficient against multidrug-resistant bacteria [49]. The main method of action of AMPs on bacterial membranes provides a more universal and less susceptible to resistance strategy than many antibiotics, which target particular bacterial functions or structures and encourage the development of resistance mechanisms [50].

### 3.2. Immunomodulatory Activity

Apart from their immediate antimicrobial actions, AMPs are important in regulating the host immunological response, which strengthens the body’s overall resistance to infections [51,52]. By boosting phagocytosis, encouraging wound healing, and neutralizing endotoxins [53], these AMPs provide further avenues for fighting infections. This versatile mechanism of action increases the effectiveness of AMPs against a variety of infections while lowering the risk of resistance development.

One of the major mechanisms by which AMPs modulate the immune system is through the enhanced recruitment of leukocytes, i.e., neutrophils, macrophages, mast cells and T cells, through the induction of chemokine release. This specific function requires the involvement of several cellular receptors, such as chemokine receptors (e.g., CCR6, CCR2), GPCRs and Toll-like receptors (TLRs). Alternatively, AMPs can affect neutrophil function by stimulating the secretion of neutrophil chemokines, such as interleukin-8 (IL-8) and (growth-regulated oncogene)-α (Gro-α or CXCL1), or the release of neutrophil extracellular traps (NETs). For example, the AMP LL-37 has been shown to induce the release of neutrophil antimicrobial granule components, including four different human α-defensins, namely human neutrophil peptide 1 (HNP1), HNP2, HNP3 and HNP4. LL-37 can also promote the formation of NETs, thereby helping to fight off viral infections [54] as well as other pathogens.

The thorough knowledge of AMP mechanisms, which include immunomodulation, intracellular targeting, and membrane disruption, emphasizes their significance in the creation of new therapeutic approaches against MDR bacteria and supports further study and the incorporation of AMPs into antimicrobial therapies [55].

### 3.3. The Synergy with Conventional Antibiotics

Moreover, it has been discovered that certain AMPs work in concert with conventional antibiotics to improve pathogen clearance by influencing the immune system in addition to lowering bacterial defenses and allowing drug access [56]. By fusing the distinct activities of AMPs with conventional antimicrobial drugs, this synergistic potential increases the effectiveness of already available antibiotics and presents a viable tactic to battle MDR infections [54].

And the possibility of synergistic effects when combined with antibiotics creates new therapeutic options and may even bring existing medications back to life and increase their clinical usefulness [57]. One study combined antimicrobial peptides tagged with the large non-natural amino acid β-naphthyl alanine (Nal) at their N or C terminus in order to increase and/or synergize the effectiveness of conventional antibiotics. The synergy between the parent peptide and the Nal-labeled peptide was assessed using the checkerboard method. Finally, these Nal-tagged antimicrobial peptides decreased the release of lipopolysaccharides in Gram-negative bacteria produced by antibiotics by over 99.95% [58].

### 3.4. Intracellular Mode of Action

AMPs also showed a variety of intracellular activities, which increased their antibacterial efficacy [59]. Certain AMPs can interact with intracellular targets by translocating across bacterial membranes without immediately disrupting the membrane [60]. Once inside the cell, these AMPs have the ability to block important functions such the creation of proteins, enzyme activity, and cell walls, which can cause internal bacterial mortality. Their membrane-disrupting actions are complemented by this intracellular mode of action, providing a dual attack that can be especially potent against bacteria that have become resistant to antibiotics that target particular cellular functions [61]. The capacity of AMPs to disrupt both membrane integrity and intracellular pathways highlights their promise as diverse antimicrobial agents capable of overcoming the advanced resistance mechanisms used by MDR bacteria.

## 4. AMPs in Overcoming Specific MDR Pathogens

AMPs have been shown application potential against the infection of drug-resistant pathogens. Furthermore, many clinical trials are currently underway to evaluate the efficacy of AMPs. For example, Magainin has entered phase III clinical trials. As one of the more in-depth studies of antimicrobial peptides, it has antibacterial activity against a variety of bacteria. There is also a PL-18 polypeptide suppository, which is a non-antibiotic anti-infective drug and has a unique antifungal and antibacterial mechanism. Many investigations and clinical trials have demonstrated the effectiveness of AMPs against a variety of MDR infections, positioning AMPs as formidable opponents with potential for therapeutic use [62]. MRSA, VRE, and MDR-TB are three of the most studied multidrug-resistant organisms (Table 3). These three species pose serious obstacles to the existing antibiotic therapies [63]. For example, MRSA has demonstrated sensitivity to many AMPs, which damage its cell membrane and restrict its growth more efficiently than certain conventional antibiotics. MRSA is a common cause of hospital-acquired illnesses [64]. Similarly, AMPs that are able to circumvent the vancomycin resistance mechanisms of VRE, another well-known hospital infection, have been directed at it. AMPs present a unique method of action that may be able to circumvent the bacterium’s intricate resistance mechanisms in the context of MDR-TB, which is a worldwide public health emergency with few available treatment alternatives. These case studies show not only AMPs’ broad-spectrum action but also their specialized efficacy against some of the most difficult MDR infections, supporting the premise that AMPs could significantly contribute to the arsenal against antibiotic-resistant illnesses.

The positive results of preclinical research have opened the door for clinical trials assessing the effectiveness and safety of AMPs in human subjects. Gradually, research results have shown that AMPs can be designed for less toxicity and increased specificity, resolving some of the early reservations about their therapeutic application. For instance, AMP-based topical formulations have demonstrated encouraging outcomes in clinical studies treating MRSA-infected wounds, including notable decreases in bacterial load and enhancements in wound healing [65]. Similar to this, inhalable AMPs are being investigated as possible therapies for MDR-TB-related lung infections, providing a focused method enabling these peptides to reach the infection site [66]. These clinical initiatives represent significant advancements in the clinical management of infections brought on by multidrug-resistant organisms as well as a validation of the results obtained from in vitro and animal models [67]. As studies go on, AMPs’ ongoing development together with thorough clinical assessment will be essential to removing obstacles to their general application and reaching their full potential as an innovative class of antibiotics.

## 5. Challenges and Limitations in AMP Utilization

AMPs exhibit encouraging therapeutic promise; nonetheless, their full potential must be realized due to a number of obstacles and constraints in their application. Concerns about toxicity and stability are two of the main barriers to AMP utilization. Since AMPs are peptides, their effectiveness may be greatly diminished in biological settings due to proteolytic breakdown [68]. Furthermore, there is a delicate balance between the antimicrobial action of AMPs and their cytotoxicity to host cells even though they specifically target microbial cells. Toxicological problems may arise from AMPs’ unfavorable interactions with host cell membranes caused by high quantities or poorly designed sequences. In order to address these issues, AMP sequences must be improved for stability and specificity. This can be accomplished by using techniques like cyclization, D-amino acid substitution, or the use of peptidomimetics, which function similarly to AMPs but are less hazardous and more stable [69].

Another major issue with AMPs is the possibility of resistance developing [70]. Compared to conventional antibiotics, AMPs’ broad-spectrum, multi-targeted mechanism of action lessens the chance of resistance, but it cannot be completely ruled out. Bacteria have developed defense mechanisms against AMPs, such as increasing the amount of proteases that break down AMPs or altering the charge of their membrane to repel cationic peptides [71]. To keep ahead of possible resistance, ongoing surveillance and study into bacterial resistance mechanisms are crucial. Additionally, resistance development can be minimized by creating AMPs that target conserved and important bacterial components [72,73]. These tactics, along with the use of AMPs in combination therapy, can support the long-term maintenance of their efficacy.

Expense and production scalability are additional challenges to the broad use of AMPs. Even though peptide chemical synthesis is flexible and can yield high-purity AMPs, it can be costly, particularly for long or complex peptides [74]. Recombinant DNA technology in biotechnological manufacturing provides an alternative, but it also has its own set of difficulties, such as making sure the peptides are correctly folded and processed. Technological developments in fermentation and synthetic biology may offer answers to these problems, increasing the viability and economy of large-scale manufacturing. Notwithstanding these obstacles, further research and development activities focused on improving AMP synthesis techniques are essential to ensuring that AMP-based treatments are a competitive alternative in the fight against MDR infections [39].

To effectively tackle these obstacles, a comprehensive strategy must be implemented, utilizing developments in peptide chemistry, biotechnology, and the comprehension of microbial pathogenesis [75]. To fully realize the therapeutic promise of AMPs, new research and teamwork will be essential in resolving these constraints as the area develops.

## 6. Advanced Strategies in AMP Enhancement and Delivery

Peptide engineering’s cutting-edge techniques have proven crucial in the fight against the difficulties that arise with using AMPs in clinical settings [76]. Changes to AMP structures and sequences are made with great care to increase potency and stability. The short peptide was designed by fusing the α-helix and β-turn sequence motifs in the symmetrical end template. The results showed that the designed peptides PQ and PP tended to form an α-helix structure after interacting with the simulated membrane environment. Most of their activities are maintained in the presence of different conditions (salt, serum, heat, pH), indicating their stability in vivo [77].

At the forefront of this innovation are methods like the construction of amphipathic peptides for better microbe targeting without damaging host cells, cyclization to minimize proteolytic breakdown, and the insertion of non-natural amino acids [78]. Furthermore, sequence optimization for enhanced interaction with microbial membranes, peptide behavior prediction, and cytotoxic effect minimization are all greatly aided by bioinformatics and computer modeling [79,80]. These modified peptides mark a substantial advancement in the creation of AMP-based treatments since they not only show enhanced resistance to enzymatic degradation but also preserve or improve their antibacterial activity.

When it comes to AMP delivery, nanotechnology has been a game-changer, solving problems with stability, specificity, and systemic toxicity [81,82,83]. Liposomes, polymeric nanoparticles, and metallic nanoparticles are examples of nanocarriers that are designed to encapsulate antimicrobial proteins (AMPs) in order to shield them from premature destruction and enable targeted distribution to infection areas. By preventing hydrolysis, boosting absorption, and avoiding efflux pumps to overcome barriers to antibiotic resistance, nanodrug delivery devices give outdated drugs new life. For instance, due to their high drug-loading capacity, adjustable physicochemical characteristics, and biocompatibility, mesoporous silica nanoparticles (MSNs) are among the most extensively researched medications as antibiotic carriers. By changing the surface characteristics, modifying the form, and decreasing the size, MSNs can greatly enhance the delivery of drugs to bacteria. Furthermore, by regulating the release of metal ions or raising reactive oxygen species, MSNs hybridized with metal ions or metal nanoparticles have more potent antibacterial actions. Additionally, antibiotics can be loaded into metal-capped MSNs to produce a synergistic antibacterial action.

This focused strategy increases the therapeutic index of AMPs while reducing off-target effects. Furthermore, nanotechnology ensures long-term antibacterial effectiveness by enabling the controlled release of AMPs [84]. The specificity of AMP distribution is further improved by innovations like the surface modification of nanoparticles to identify particular bacterial markers, illustrating how nanotechnology has the ability to completely transform the way AMP medicines are administered.

AMPs can have synergistic effects when combined with other antimicrobial drugs or treatments, providing a tactical advantage against multidrug-resistant bacteria [85]. In the early phases of antimicrobial drug discovery, the synergistic combination of antibiotics and adjuvants with distinct properties provides a significant but mainly unexplored way to “reutilize” already existing biomaterials while addressing concerns of potency, spectrum, toxicity, and resistance. Combining broad-spectrum antimicrobial lipopeptides, which are intended to improve intracellular accumulation efficiency and membrane targeting, with antibiotics increases antibacterial efficacy. This method makes use of AMPs’ distinct modes of action to boost the effects of conventional antibiotics, overcome bacterial resistance, and alter the human immunological response [86]. Promising outcomes from combination therapy research include decreased bacterial load, improved infection clearance, and inhibition of the emergence of resistance [87]. In order to create more potent, broad-spectrum treatment regimens, researchers carefully consider the complementary activities of AMPs and other antimicrobials when combining different substances. In order to address the growing problem of antibiotic resistance, combination medicines are being investigated. Clinically related multidrug resistance mutations increase the sensitivity of bacteria to antimicrobial peptides, and the incidental sensitivity of multidrug-resistant bacteria is partly due to the regulation of changes in the composition of bacterial outer membrane lipopolysaccharide.

These advances allow the identification of antimicrobial peptide–antibiotic combinations that enhance the activity of antibiotics against multidrug-resistant bacteria and slow the de novo evolution of drug resistance [88,89]. In particular, when administered together as an adjuvant, the antimicrobial peptide glycine-leucine-amide causes the antibiotic resistance level of drug-resistant bacteria to be reduced by up to 30 times. This emphasizes the value of a multidisciplinary approach that incorporates knowledge from clinical medicine, pharmacology, and microbiology. These cutting-edge AMP delivery and augmentation techniques are an excellent example of the creative methods being used to realize the full potential of AMPs in antimicrobial therapy [84]. The field is closer to realizing the potential of AMPs as a cornerstone in the fight against infectious diseases, particularly those caused by MDR pathogens [90]. This is due to efforts to address the inherent limitations of AMPs through peptide engineering, utilize nanotechnology for targeted delivery, and investigate synergistic combination therapies [91].

## 7. Regulatory and Ethical Considerations

AMPs must navigate a challenging terrain of clinical trials and regulatory barriers in order to go from encouraging laboratory results to licensed medicinal treatments [92]. Numerous studies are being conducted to evaluate the safety, pharmacokinetics, and efficacy of AMPs in humans, which is a testament to their potential. AMPs are currently undergoing clinical trials. These trials include a wide range of applications, including systemic therapy for drug-resistant bacterial and fungal infections as well as topical treatments for wound healing and bacterial infections [93,94]. But moving from preclinical success to clinical efficacy is not easy. It takes thorough testing to satisfy the exacting standards imposed by regulatory agencies like the European Medicines Agency (EMA) and the Food and Drug Administration (FDA) in the United States. By ensuring that only AMPs with a strong safety record, noteworthy therapeutic effects, and reliable manufacturing quality are approved for sale, the procedure protects patient safety and preserves the credibility of the medical community.

Due to their distinct modes of action and variety of biological sources, AMPs provide special obstacles when navigating the regulatory road for approval. Comprehensive information on the AMPs’ mode of action, toxicity, potential for developing resistance, and interactions with other medications is needed by regulatory bodies. However, obtaining this information can be difficult and expensive. The regulatory approval process is made more difficult by the intrinsic diversity of peptide sequences and the possibility of immunogenic reactions. Furthermore, the absence of well-established regulatory frameworks designed especially for peptide-based medicines may cause ambiguities and prolong the licensing process. In order to overcome these obstacles, researchers, regulatory specialists, and legislators must work closely together to create peptide-specific, lucid standards that will expedite the assessment and approval of AMPs and guarantee that patients in need can receive these ground-breaking medicines.

A wide range of issues are covered by ethical considerations in the creation and application of AMPs, from guaranteeing fair access to these potentially life-saving treatments to exercising responsible use stewardship to delay the formation of resistance. When developing AMPs, the ecological impact must be taken into account, especially for peptides produced from natural sources, to make sure that methods of collecting or synthesis do not compromise ecosystem balance or biodiversity [95]. The use of AMPs in clinical settings presents ethical issues with relation to informed consent, patient selection for trials, and the effects of bringing strong antimicrobial drugs into settings where widespread antibiotic usage is already placing stress on microbial ecosystems [96]. The development of worldwide guidelines for AMP use, ethical research techniques, and open patient communication are crucial in addressing these ethical dilemmas and guaranteeing that AMP therapies provide the intended benefits while posing the fewest possible hazards to individuals and communities.

While taken as a whole, these factors show how difficult it is to strike a balance between creativity, legality, and moral obligation while developing and utilizing AMPs. The future of AMP therapy will be shaped by continuing discussions among scientists, clinicians, regulatory agencies, and society as this field of study develops, ensuring that it complies with ethical and medical norms.

## 8. Future Perspectives

AMP research is gaining ground quickly due to new trends in design and discovery that could lead to the discovery of novel therapeutic opportunities. Advancements in proteomics and genomics are making it easier to identify new AMPs from a wider range of organisms, increasing the resistance of the body against harmful microorganisms. Synthetic biology and peptide engineering developments are making it possible to customize AMPs to maximize their effectiveness, stability, and specificity while reducing their toxicity. Computational modeling and machine learning technologies support these efforts by enhancing antimicrobial activity and streamlining the design process through the prediction of peptide structure–function connections [97]. This rapidly developing field of study represents the state of biomedical science at the forefront and ushers in a new era of precision antimicrobials, which are drugs that are specifically designed to fight particular illnesses or germs, including ones that are resistant to conventional antibiotics [98].

MDR bacteria pose a threat to global health, which emphasizes the urgent need for novel solutions and establishes AMPs as a cornerstone of the collaborative effort to protect public health. Their distinct mechanisms of action and all-around effectiveness present a viable substitute for conventional antibiotics with the potential to revolutionize the way infectious diseases are treated [99]. Beyond their direct antimicrobial activities, AMPs have a wide range of roles in immune regulation and wound healing, which highlights their significance in an all-encompassing antimicrobial strategy [100]. The worldwide management of MDR infections may be greatly impacted by the introduction of AMPs into clinical practice as research into their full therapeutic potential unfolds. This could lessen the burden of antibiotic resistance and enhance patient outcomes [101]. To fully realize this promise, though, coordinated efforts are needed to address the current roadblocks in AMP development, which include stability, cost-effective production, and safety.

Future directions for AMP research and development require more money and improved international cooperation. Antimicrobial resistance is a complex issue that cuts beyond national boundaries and healthcare systems, necessitating an international response. Research on amplified mental pain can move more quickly from discovery to therapeutic application when information, resources, and technology are shared within disciplines and nations. Furthermore, significant funding from the public and private sectors is required to fund the comprehensive study, development, and clinical trials required to introduce novel AMP-based treatments to the market. The pharmaceutical sector, governments, and international health organizations need to commit to investing in the future of antimicrobial resistance agents and acknowledge their strategic role in the antimicrobial arsenal [102]. Through the establishment of a collaborative and supportive atmosphere, the international community may fully utilize AMPs to tackle one of the most urgent health issues of our day.

## 9. Conclusions

AMPs are a new and adaptable strategy for combating infections that have resisted conventional antibiotic treatments, making them a ray of hope in the ongoing fight against MDR bacteria. Their importance as a vital addition to the antimicrobial arsenal is highlighted by their broad-spectrum activity, distinct modes of action, and potential for synergistic effects with current antimicrobials. But in order to fully realize the therapeutic potential of AMPs, more research and development are needed to address issues with toxicity, stability, resistance, and the scalability of production. As the world’s health community struggles to contain the growing threat of antibiotic resistance, developing AMPs via creative research, interdisciplinary teamwork, and consistent funding becomes an essential tactic. Anticipating the future, the incorporation of AMPs into antimicrobial therapy presents a promising avenue to not only overcome present treatment barriers for multidrug-resistant infections but also to help shape a future in which the prevalence of antibiotic resistance is considerably reduced, protecting global public health outcomes.

## Figures and Tables

**Table 1:** The source and function of AMPs.

AMP	Source	Function	Reference
defensins	human	innate effectors and immune modulators	[36]
cathelicidins	human	broad-spectrum antimicrobial activity and immunomodulatory properties	[37]
Magainin	frog	kill microbes and prevent infection	[38]
thionine	plant	a rich repertoire of antimicrobial properties	[39]

**Table 2:** The latest status of the design of new AMPs or peptide mimics.

New AMPs or Peptide Mimics	Main Contents	Reference
radial amphiphilic antimicrobial peptides (RAPs)	It can recognize bacterial phospholipid PG with high selectivity. Through a variety of experimental techniques, the interaction and recognition mechanism between AMP and PG were revealed, and the key role of helix structure and hydrophobic region hiding in recognition was clarified. It introduced a pattern of targeting bacterial-specific phospholipids for the development of antimicrobial peptides with high bacterial selectivity.	[43]
HR2-7	It had strong antibacterial activity against a variety of Gram-positive and negative bacteria and plant pathogenic fungi, and it showed a good control effect on related diseases on a variety of crops.	[44]
Ni-IH-7	It has phospholipase c-like activity and peroxidase-like activity. It can target and bind mannan on the surface of Candida albicans as well as induce lipid peroxidation to cause ferroptosis and hydrolysis of glycerophospholipids, thereby rapidly killing fungi. It has a specific fungicidal function and stable bactericidal performance, which provides ideas for the development of new antibacterial drugs.	[45]
Pyrgos(n)cages	It has the characteristics of dense and evenly distributed positive charge, highly rigid structure, and balance of hydrophobicity and hydrophilicity. It has a strong antibacterial effect on Gram-positive bacteria, especially clinical multidrug-resistant bacteria. The bactericidal mechanism involves the dual effects of cell membrane and DNA. It shows excellent antibacterial activity in both intracellular and mouse models, and it provides new ideas for combating intracellular bacteria and drug-resistant bacteria.	[46]

**Table 3:** The contribution of antimicrobial peptides against MRSA, VRE and MDR-TB, the three most studied multidrug-resistant bacteria.

AMPs	MDR Bacteria	Mode of Action	Reference
Clavanin B	MRSA	the protein–peptide interaction of the MRSA target proteins, Penicillin Binding Protein 2a and Panton–Valentine leukocidin toxin, with the antimicrobial peptide Clavanin B	[7]
Ceragenins (CSA)	VRE	interact with the cytoplasmic membrane to exert an antibacterial effect	[8]
Defensins	MDR-TB	resisting the invasion of microorganisms and regulating the immune response	[9]
